# Jasmonate: the Swiss army knife in the plant’s pocket

**DOI:** 10.1093/jxb/erac511

**Published:** 2023-02-13

**Authors:** Ziqiang Zhu

**Affiliations:** College of Life Science, Nanjing Normal University, Nanjing 210023, China

**Keywords:** Defense response, flowering, jasmonate, nutrient deficiency, plant hormone, seed germination, regeneration, root hair, wounding

## Abstract

Jasmonate is a well-known defence hormone for plants, but it is also necessary for growth and development. Indeed, the identification of the COI1 receptor was based on the jasmonate-triggered response of root growth inhibition. In this special issue, a collection of review papers and two research papers discuss the current state of progress in this field, covering areas from seed germination and flowering to the *Jasminum sambac* genome.

Jasmonate is widely recognized as a defence hormone for plants against attack by insects and necrotrophic fungi (Zhang *et al.*, 2017), but it also controls growth and development. For example, plants defective in jasmonate biosynthesis or signaling are male-sterile (Xie *et al.*, 1998; Stintzi and Browse, 2000). Jasmonate treatment inhibits root elongation, and this facilitates forward genetics studies: as a result of this, two important components in jasmonate signaling have been uncovered. The first is COI1, which was identified via the jasmonate-triggered response of inhibition of root growth (Feys *et al.*, 1994), and is an F-box protein that links jasmonate signalling and fertility to the SCF^COI1^ E3 ubiquitin–ligase complex (Xie *et al.*, 1998; Devoto *et al.*, 2002), and the second is the transcription factor MYC2, which participates in jasmonate signaling (Lorenzo *et al.*, 2004). Although extensive screening of mutants and protein–protein interaction tests were done, the relationship between COI1 and MYC2 remained unclear for many years. Then, in 2007, three separate groups individually demonstrated that JAZ-repressor proteins were the long-sought missing link between COI1 and MYC2 (Chini *et al.*, 2007; Thines *et al.*, 2007; Yan *et al.*, 2007). Without jasmonate, JAZ proteins interact with MYC2 and repress its transcriptional activity. Jasmonate (here referring to its active form JA-Ile) stimulates the interaction between COI1 and JAZ in the manner of a ‘molecular glue’ and then degrades the JAZ proteins to release their repression. This de-repression mechanism was further reinforced by the identification of a variety of JAZ-interacting transcription factors (Wasternack and Song, 2017).

In 2017, the *Journal of Experimental Botany* published a special issue to celebrate the first decade after the identification of JAZ proteins (https://academic.oup.com/jxb/issue/68/6). That issue showcased advances in research covering jasmonate biosynthesis, evolution, and signaling, its relations with transcription factors, senescence, and defenses that had occurred in the previous 10 years. Two years later, the journal collaborated with Drs Edward Farmer and Alain Goossens to publish another special issue on the topic of jasmonate (https://academic.oup.com/jxb/issue/70/13). This issue, which was associated with the Regulatory Oxylipins International Meeting (1–4 April, 2019) held in Belgium, focused on defense, signal cross-talk, and newly discovered regulatory mechanisms. Since the publication of these two special issues, there have been many new novel discoveries in the field of jasmonate research and hence the time is right to present a new collection of papers. In this issue, we focus on the roles of jasmonate in plant growth and development areas.

Seed germination is the beginning of the plant life cycle. Jasmonate coordinates seed germination through interacting with abscisic acid (ABA), gibberellin (GA), and light signaling, as reviewed by Pan *et al.* (2023). They first note that exogenous jasmonate inhibits seed germination together with ABA in a synergistic way and that the endogenous jasmonate content decreases during germination. They then highlight recent progress in the characterization of the molecular mechanisms underlying the jasmonate–ABA control of germination. JAZ1/JAZ5/JAZ8 interact with ABI5 (a transcription factor in ABA signaling) and repress its transcriptional activity. Jasmonate treatment triggers JAZ degradation and re-activates ABI5 to trigger its downstream responses. Meanwhile, ABA promotes jasmonate biosynthesis to synergistically inhibit germination. The review also highlights how GA and light affect jasmonate synthesis within the multiplex regulatory network.

Roots and root hairs are unique organs for anchoring plants in the soil and absorbing water and nutrients, and Han *et al.* (2023) review novel findings in the control of their development by jasmonate. Inhibition of root growth is a hallmark phenotype for screening of jasmonate-related mutants, and studies have shown that MYC2 is a positive regulator for this response (Lorenzo *et al.*, 2004). The review describes how the bHLH subgroup IIId transcription factors (i.e. bHLH3 and bHLH17) negatively regulate root growth inhibition through physically interacting with JAZ proteins. Jasmonate also stimulates root-hair development, and (Han *et al.* (2023) first ­introduce the research background and then highlight the recent finding that JAZ proteins interact with and inhibit the RHD6 and RSL4 transcription factors to control root-hair elongation. Previous reports have shown that two ethylene-activated transcription factors, EIN3 and EIL1, interact with JAZ proteins and participate in the regulation of root-hair growth (Zhu *et al.*, 2011). EIN3 also interacts with RHD6 and directly controls the transcription of *RSL4* (Feng *et al.*, 2017). The review neatly summarizes these aspects and proposes future directions for study in this field.

Flower development is key for angiosperm reproduction and jasmonate is crucial for stamen development. Huang *et al.* (2023) highlight recent breakthroughs in the study of jasmonate-controlled male fertility. They comprehensively describe the positive and negative players in this complicated regulatory network. Moreover, they compare these signaling modules in Arabidopsis, tomato, rice, maize, and sorghum, making this review of interest to researchers across a range of different species.

In their research paper, Cebrian *et al.* (2023) use the fruit of *Cucurbita pepo* to study the opening of male and female flowers and fruit development. They identify a mutant that is defective in jasmonate synthesis and that exhibits defective male and female flower opening and has exaggerated parthenocarpic fruit development. They also study the coordination between ethylene and jasmonate in the control of male and female flower development.

Plants have remarkable abilities to regenerate in response to wounding, and the generation of transgenic plants is one very important example of how this is exploited. Extensive studies have generally focused on auxin and cytokinin, and it is only recently that the roles of jasmonate in regeneration have become better understood. In general, wounding is the initial signal for regeneration, and this is correlated with a boost in jasmonate synthesis. In a review that will open a new window for jasmonate studies and hopefully attract more researchers to this field, Zhang *et al.* (2023) systematically review the roles of jasmonate in tissue repair, callus formation, *de novo* organ regeneration, and somatic embryogenesis. Another important question related to wounding is herbivore attack, and Gao and Farmer (2023) neatly propose an osmoelectric siphon model to account for signal and water dispersal in wounded plants.

Plants cannot survive without a nutrient supply, and Shikha *et al.* (2023) review recent advances in jasmonate-mediated responses to both macronutrient and micronutrient deficiencies. In a paper that helps shed new light on our understanding of the roles of plant hormones in nutrient uptake and transport, they also illustrate the impacts of nutrient availability on jasmonate synthesis and signaling.

Of course, defense responses has to be a topic within any special issue related to jasmonates, and in this case we include a review paper by Li *et al.* (2023) that summarizes recent advances in the study of the arm’s race between plant hosts and pathogens. This review should contribute to future plant biotechnology studies for improving crop breeding.

Last but not least, this special issue includes a research paper by Xu *et al.* (2023) that reports on the *Jasminum sambac* genome. The authors have obtained a high-quality and telomere-to-telomere assembly consensus genome of the double-petal *J. sambac* with a contig N50 of 35.96 Mb. This study also presents a genomic analysis that explains why aromatic volatile jasmonate is highly accumulated in *J. sambac* tissues.
